# Clinical phenotypes and outcomes associated with SARS-CoV-2 Omicron variants BA.2, BA.5 and BQ.1.1 in critically ill patients with COVID-19: a prospective, multicenter cohort study

**DOI:** 10.1186/s40635-023-00536-0

**Published:** 2023-08-07

**Authors:** Nicolas de Prost, Etienne Audureau, Sébastien Préau, Raphaël Favory, Aurélie Guigon, Pierre Bay, Nicholas Heming, Elyanne Gault, Tài Pham, Amal Chaghouri, Guillaume Voiriot, Laurence Morand-Joubert, Sébastien Jochmans, Aurélia Pitsch, Sylvie Meireles, Damien Contou, Amandine Henry, Adrien Joseph, Marie-Laure Chaix, Fabrice Uhel, Diane Descamps, Malo Emery, Claudio Garcia-Sanchez, Charles-Edouard Luyt, Stéphane Marot, Frédéric Pène, Anne-Sophie Lhonneur, Stéphane Gaudry, Ségolène Brichler, Lucile Picard, Armand Mekontso Dessap, Christophe Rodriguez, Jean-Michel Pawlotsky, Slim Fourati, the SEVARVIR investigators, Keyvan Razazi, Raphaël Bellaïche, Elie Azoulay, Jean-François Timsit, Matthieu Turpin, Nina de Montmollin, Julien Mayaux, Damien Roux, Djillali Annane, Cédric Hartard, Antoine Kimmoun, Ferhat Meziani, Louis-Marie Jandeaux, Samira Fafi-Kremer

**Affiliations:** 1 Service de Médecine Intensive Réanimation, Hôpitaux Universitaires Henri Mondor, Assistance Publique, Hôpitaux de Paris (AP-HP), Créteil, France; 2 Groupe de Recherche Clinique CARMAS, Université Paris-Est-Créteil (UPEC), Créteil, France; 3 Université Paris-Est-Créteil (UPEC), Créteil, France; 4 Department of Public Health, Hôpitaux Universitaires Henri Mondor, Assistance Publique, Hôpitaux de Paris (AP-HP), Créteil, France; 5 IMRB INSERM U955, Team CEpiA, Créteil, France; 6 U1167, RID-AGE Facteurs de Risque et Déterminants Moléculaires des Maladies Liées au Vieillissement, University Lille, Inserm, CHU Lille, Institut Pasteur de Lille, 59000 Lille, France; 7 Service de Virologie, CHU de Lille, 59000 Lille, France; 8 Médecine Intensive Réanimation, Hôpital Raymond Poincaré, Assistance Publique, Hôpitaux de Paris (AP-HP), Garches, France; 9 Laboratoire de Virologie, Hôpital Ambroise Paré, Assistance Publique, Hôpitaux de Paris (AP-HP), Boulogne, France; 10 Service de Médecine Intensive-Réanimation, Assistance Publique, Hôpitaux de Paris, Hôpital de Bicêtre, DMU 4 CORREVE Maladies du Cœur et Des Vaisseaux, FHU Sepsis, Le Kremlin-Bicêtre, France; 11 Inserm U1018, Equipe d’Epidémiologie Respiratoire Intégrative, CESP, 94807 Villejuif, France; 12 Laboratoire de Virologie, Hôpital Paul Brousse, Assistance Publique, Hôpitaux de Paris, Villejuif, France; 13 Sorbonne Université, Centre de Recherche Saint-Antoine INSERM, Médecine Intensive Réanimation, Hôpital Tenon, Assistance Publique, Hôpitaux de Paris, Paris, France; 14 Sorbonne Université, INSERM, Institut Pierre Louis d’Epidémiologie et de Santé Publique, Paris, France; 15 Laboratoire de Virologie, Hôpital Saint-Antoine, Assistance Publique, Hôpitaux de Paris, 75012 Paris, France; 16 Service de Réanimation Polyvalente, Hôpital Marc Jacquet, Melun, France; 17 Laboratoire de Microbiologie, Hôpital Marc Jacquet, Melun, France; 18 Service de Réanimation Médico-Chirurgicale, Assistance Publique, Hôpitaux de Paris, Hôpital Ambroise Paré, Boulogne, France; 19 Service de Réanimation, Hôpital Victor Dupouy, Argenteuil, France; 20 Service de Virologie, Hôpital Victor Dupouy, Argenteuil, France; 21 Médecine Intensive Réanimation, Hôpital Saint-Louis, Assistance Publique, Hôpitaux de Paris, Paris, France; 22 Université de Paris, Inserm HIPI, 75010 Paris, France; 23 Laboratoire de Virologie, Hôpital Saint-Louis, Assistance Publique, Hôpitaux de Paris, 75010 Paris, France; 24 n, Université de Paris, APHP, Hôpital Louis Mourier, DMU ESPRIT, Service de Médecine Intensive Réanimatio, Colombes, France; 25 INSERM U1151, CNRS UMR 8253, Institut Necker-Enfants Malades (INEM), Department of Immunology, Infectiology and Hematology, Paris, France; 26 Université de Paris, IAME INSERM UMR 1137, Service de Virologie, Hôpital Bichat-Claude Bernard, Assistance Publique, Hôpitaux de Paris, Paris, France; 27 Service de Réanimation, Hôpital Saint-Camille, Bry-Sur-Marne, France; 28 Laboratoire de Biologie, Hôpital Saint-Camille, Bry-Sur-Marne, France; 29 Sorbonne Université, Assistance Publique, Hôpitaux de Paris, Hôpital Pitié–Salpêtrière, Médecine Intensive Réanimation, Paris, France; 30 INSERM UMRS_1166-iCAN, Institute of Cardiometabolism and Nutrition, Paris, France; 31 Département de Virologie, Hôpital Pitié–Salpêtrière, Assistance Publique-Hôpitaux de Paris (AP-HP), Paris, France; 32 Médecine Intensive Réanimation, Hôpital Cochin, Assistance Publique, Hôpitaux de Paris, Paris, France; 33 Laboratoire de Virologie, Hôpital Cochin, Assistance Publique, Hôpitaux de Paris, Paris, France; 34 Service de Réanimation, Hôpital Avicenne, Assistance Publique, Hôpitaux de Paris, Bobigny, France; 35 Laboratoire de Virologie, Hôpital Avicenne, Assistance Publique, Hôpitaux de Paris, Bobigny, France; 36 Département d’Anesthésie Réanimations Chirurgicales, Hôpitaux Universitaires Henri Mondor, Assistance Publique, Hôpitaux de Paris (AP-HP), Créteil, France; 37 Department of Virology, Hôpitaux Universitaires Henri Mondor, Assistance Publique, Hôpitaux de Paris, Créteil, France; 38 INSERM U955, Team “Viruses, Hepatology, Cancer”, Créteil, France

**Keywords:** SARS-CoV-2, Omicron, Sublineage, COVID-19, Acute respiratory failure

## Abstract

**Background:**

Despite current broad natural and vaccine-induced protection, a substantial number of patients infected with emerging SARS-CoV-2 variants (e.g., BF.7 and BQ.1.1) still experience severe COVID-19. Real-life studies investigating the impact of these variants on clinical outcomes of severe cases are currently not available. We performed a prospective multicenter observational cohort study. Adult patients with acute respiratory failure admitted between December 7, 2021 and December 15, 2022, in one of the 20 participating intensive care units (17 from the Greater Paris area and 3 from the North of France) were eligible for inclusion if they had SARS-CoV-2 infection confirmed by a positive reverse transcriptase-polymerase chain reaction (RT-PCR). Full-length SARS-CoV-2 genomes from all included patients were sequenced by means of next-generation sequencing. The primary endpoint of the study was day-28 mortality.

**Results:**

The study included 158 patients infected with three groups of Omicron sublineages, including (i) BA.2 variants and their early sublineages referred as “BA.2” (n = 50), (ii) early BA.4 and BA.5 sublineages (including BA.5.1 and BA.5.2, n = 61) referred as “BA.4/BA.5”, and (iii) recent emerging BA.5 sublineages (including BQ.1, BQ.1.1, BF.7, BE.1 and CE.1, n = 47) referred as “BQ.1.1”. The clinical phenotype of BQ1.1-infected patients compared to earlier BA.2 and BA.4/BA.5 sublineages, showed more frequent obesity and less frequent immunosuppression. There was no significant difference between Omicron sublineage groups regarding the severity of the disease at ICU admission, need for organ failure support during ICU stay, nor day 28 mortality (21.7%, n = 10/47 in BQ.1.1 group vs 26.7%, n = 16/61 in BA.4/BA.5 vs 22.0%, n = 11/50 in BA.2, p = 0.791). No significant relationship was found between any SARS-CoV-2 substitution and/or deletion on the one hand and survival on the other hand over hospital follow-up.

**Conclusions:**

Critically-ill patients with Omicron BQ.1.1 infection showed a different clinical phenotype than other patients infected with earlier Omicron sublineage but no day-28 mortality difference.

**Supplementary Information:**

The online version contains supplementary material available at 10.1186/s40635-023-00536-0.

## Background

Since summer 2022, an unprecedented diversification of SARS-CoV-2 Omicron variant sublineages has followed the emergence and global spread of Omicron lineage BA.2 and subsequently BA.5. Currently, BF.7, followed by BQ.1.1 and the XBB.1.5 recombinant appear to be among the fastest growing variants in the world [1]. BF.7 is also one of the dominant circulating variants in China since restrictions policies were lifted in the country at the end of 2022 [2]. These variants differ from the original BA.2 and BA.5 variants by several amino acid substitutions in the receptor-binding domain (RBD) of the Spike protein that induce key antigenic shifts with altered antibody evasion properties [3, 4].

Although infection with the Omicron variant was recently demonstrated to yield less severe disease than other variants in critically ill patients [5], the relationship between Omicron sublineages and the severity of COVID-19 is not well understood. Preliminary animal model studies suggest that some BA.2 descendants, such as BA.5 and BA.2.75, might cause more severe disease than their parental BA.2 [6, 7]. Conversely, the intrinsic pathogenicity of variant BQ.1.1 was reported to be equivalent or reduced, as compared to that of BA.5 in a hamster model [8]. No real-life studies investigating the relationship between the newly emerging SARS-CoV-2 variants, including BF.7 and BQ.1.1, and clinical outcomes have been reported. During the first variant Omicron wave in early 2022, almost 50% of patients admitted to the intensive care unit (ICU) for acute respiratory failure were immunocompromised and they had a poor antibody response to vaccination [9]. The more recent BA.2 and BA.5 Omicron sublineages (e.g., BF.7, BQ.1.1, and XBB) have shown in vitro resistance to monoclonal antibodies [10], a treatment option for immunocompromised patients with severe COVID-19 [11]. Whether emerging substitutions conferring resistance to monoclonal antibodies are associated with different clinical outcomes has not yet been investigated.

We hypothesized that emerging Omicron sublineages could be associated with more severe COVID-19 and different clinical presentation in critically ill patients. In the present study, we thus compared the characteristics of critically ill patients with acute respiratory failure infected with the latest emerging SARS-CoV-2 sublineages circulating in France in autumn and winter 2022, including BF.7 and BQ.1.1 shown to have acquired antibody neutralization escape capacity [10, 12, 13] referred as “BQ.1.1” group, with those of patients infected with earlier BA.2 referred as “BA.2”, and earlier BA.4 and BA.5 variants referred as “BA.4/BA.5”. The main objective of this study was to determine the association between Omicron sublineages, categorized into three groups (i.e., BA.2, BA.4/BA.5, and BQ.1.1 groups), and day-28 mortality. Secondary objectives were to explore the association (1) between Omicron sublineages and clinical features upon ICU admission and outcomes during ICU stay; and (2) between specific viral mutations/mutational patterns and day 28 mortality.

## Patients and methods

### Study design and patients

The current study is a substudy of the SEVARVIR study. SEVARVIR is a prospective multicenter observational cohort study. Patients admitted between December 7, 2021 and December 15, 2022, in one of the 20 participating ICUs (17 from the Greater Paris area and 3 from the North of France, see Additional file 1: Table S1 for the list of participating centers) were eligible for inclusion in the SEVARVIR cohort study (NCT05162508) if they presented with the following inclusion criteria: age ≥ 18 years, SARS-CoV-2 infection confirmed by a positive reverse transcriptase-polymerase chain reaction (RT-PCR) in nasopharyngeal swab samples, admission in the ICU for acute respiratory failure (i.e., peripheral oxygen saturation (SpO_2_) ≤ 90% and need for supplemental oxygen or any kind of ventilator support, including high flow oxygen therapy, continuous-positive airway pressure, and non-invasive or invasive mechanical ventilation), patient or next of kin informed of study inclusion. Patients with SARS-CoV-2 infection but no acute respiratory failure or with a RT-PCR cycle threshold (Ct) value > 32 in nasopharyngeal swabs were not included. The study was approved by the Comité de Protection des Personnes Sud-Méditerranée I (N° EudraCT/ID-RCB: 2021-A02914-37). Informed consent was obtained from all patients or their relatives.

**Fig. 1:**
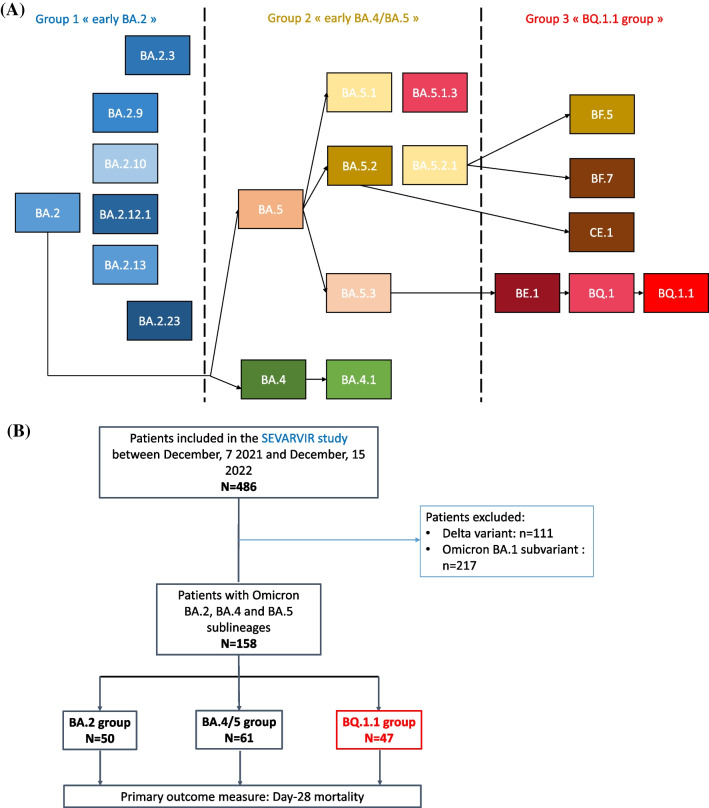
**A** Diagram representing all SARS-CoV-2 Omicron lineages included in the study (starting from BA.2 and their descendant sublineages), as designated by PANGOLIN (https://cov-lineages.org/). **B** Study flow chart. A total of 486 patients were included in the SEVARVIR study between December, 7 2021 and December, 15 2022, with 158 patients infected with BA.2, BA.4, and BA.5 Omicron sublineages, who were included in this substudy

For this substudy focused on BA.2, BA.4 and BA.5-infected patients (including sublineage BQ.1.1), we decided to stop the inclusion period when the BQ.1.1 sublineage epidemic dynamic started to decrease in France (i.e., week 51, 2022). The inclusion period started when the first patients infected with the BA.2 Omicron sublineage, which we thought was the most relevant control group, were detected (i.e., week 12, 2021). Omicron sublineages were categorized into three groups: “BA.2”, “BA.4/BA.5” and “BQ.1.1 group” (Fig. 1A).

Demographics, clinical and laboratory variables were recorded upon ICU admission and during ICU stay. Patients’ frailty was assessed using the Clinical Frailty Scale [14]. The severity of the disease upon ICU admission was assessed using the World Health Organization (WHO) 10-point ordinal scale [15], the sequential organ failure assessment (SOFA [16]) score, and the simplified acute physiology score (SAPS) II score [17]. Acute respiratory distress syndrome (ARDS) was defined according to the Berlin definition [18]. Ventilator-acquired pneumonia was defined according to current French guidelines [19]. COVID-19-associated pulmonary aspergillosis (CAPA) was defined according to ECMM/ISHAM consensus criteria [20]. Immunosuppression was defined as solid-organ transplant, active onco-hematological malignancy (within the past three years), HIV infection, long-term corticosteroid treatment (i.e., more than three months of > 0.5 mg/kg/day prednisone equivalent), and exposure to any other immunosuppressive treatment. Obesity was defined as body mass index > 30 kg/m^2^.

The primary endpoint of the study was day-28 mortality. Secondary endpoints included need for invasive mechanical ventilation during ICU stay, number of live ventilator-free days at day 28, and need for extracorporeal membrane oxygenation support (ECMO) during ICU stay.

### SARS-CoV-2 variant determination

The full-length SARS-CoV-2 genomes were sequenced by means of next-generation sequencing. Briefly, viral RNA was extracted from nasopharyngeal swabs in viral transport medium using NucliSENS^®^ easyMAG kit on EMAG device (bioMérieux, Marcy-l’Étoile, France). Sequencing was performed with the Illumina COVIDSeq Test (Illumina, San Diego, California), that uses 98-target multiplex amplifications along the full SARS-CoV-2 genome [21]. The libraries were sequenced with NextSeq 500/550 High Output Kit v2.5 (75 Cycles) on a NextSeq 500 device (Illumina). The sequences were demultiplexed and assembled as full-length genomes by means of the DRAGEN COVIDSeq Test Pipeline on a local DRAGEN server (Illumina). Lineages and clades were interpreted using Pangolin and NextClade [22]. For mutational pattern analysis at the amino acid level, only high-quality sequences, i.e., sequences covering ≥ 90% of nucleotides of the full-length viral genome and 95% of the spike gene, were considered.

Key amino acid substitutions in spike in the BQ.1.1 group spike were defined compared to its direct progenitor BA.5. BQ.1.1 has indeed some additional spike mutations in some key antigenic sites, which confer further immune escape ability over pre-existing lineages (e.g., deletion (Del)69/70, Del140, other N-terminal domain amino acid mutations, R346T/I, K444T/R, L4552R, N460K, A484T/V, F486V, other S1/Receptor Binding Domain substitutions and S2 substitutions). Full-length viral genome sequence analysis yielding high coverage have been deposited in Genbank (GenBank accession numbers OQ423331-OQ423468; https://www.ncbi.nlm.nih.gov/genbank/).

### Statistical analysis

Descriptive results are presented as mean (± standard deviation [SD]) or median (1st–3rd quartiles) for continuous variables, and as numbers with percentages for categorical variables. Two-tailed p-values < 0.05 were considered statistically significant. Unadjusted comparisons between patients infected with three groups of Omicron sublineages defined a priori (including parental early BA.2 sublineages, early BA.4 and BA.5 sublineages, and emerging BA.5 sublineages harboring resistance-associated substitutions to available monoclonal antibodies, including BQ.1, BQ.1.1, BF.7, BE.1 and CE.1 assigned as the “BQ.1.1 group”) were performed using Chi square or Fisher’s exact tests for categorical variables, and ANOVA or Kruskal–Wallis tests for continuous variables, as appropriate. We also performed a sensitivity analysis on BQ.1.1 sublineage, excluding BF.7 and BE.1 sublineages and comparing BQ.1.1-infected patients to the two first groups (BA.2- and early BA.4/BA.5-infected patients) because BQ.1.1 was one of the most prevalent sublineage circulating in France and the world during fall 2022 [23, 24]. Missing data were not imputed. The overall sample size of the SEVARVIR study was a priori defined (n = 2000). The sample size of this substudy was not predefined. Indeed, we had anticipated that data could be sequentially extracted from the prospective database based on epidemiological surges. Results have been reported according to the STROBE guidelines for cohort studies.

Adjusted analyses of the association between Omicron sublineages and 28-day mortality relied on multivariable logistic regression models, entering variables previously shown to be important confounding factors, including age, gender, SOFA score and immunosuppression. Adjusted odds ratios (aOR) along with their 95% confidence intervals (CI) were computed. An exploratory evaluation of the associations between hospital mortality and results from mutational pattern analysis at the amino acid level was performed by unadjusted Cox proportional hazard regression modeling.

Analyses were performed using Stata V16.1 statistical software (StataCorp, College Station, TX, USA), and R 4.2.0 (R Foundation for Statistical Computing, Vienna, Austria).

## Results

**Fig. 2:**
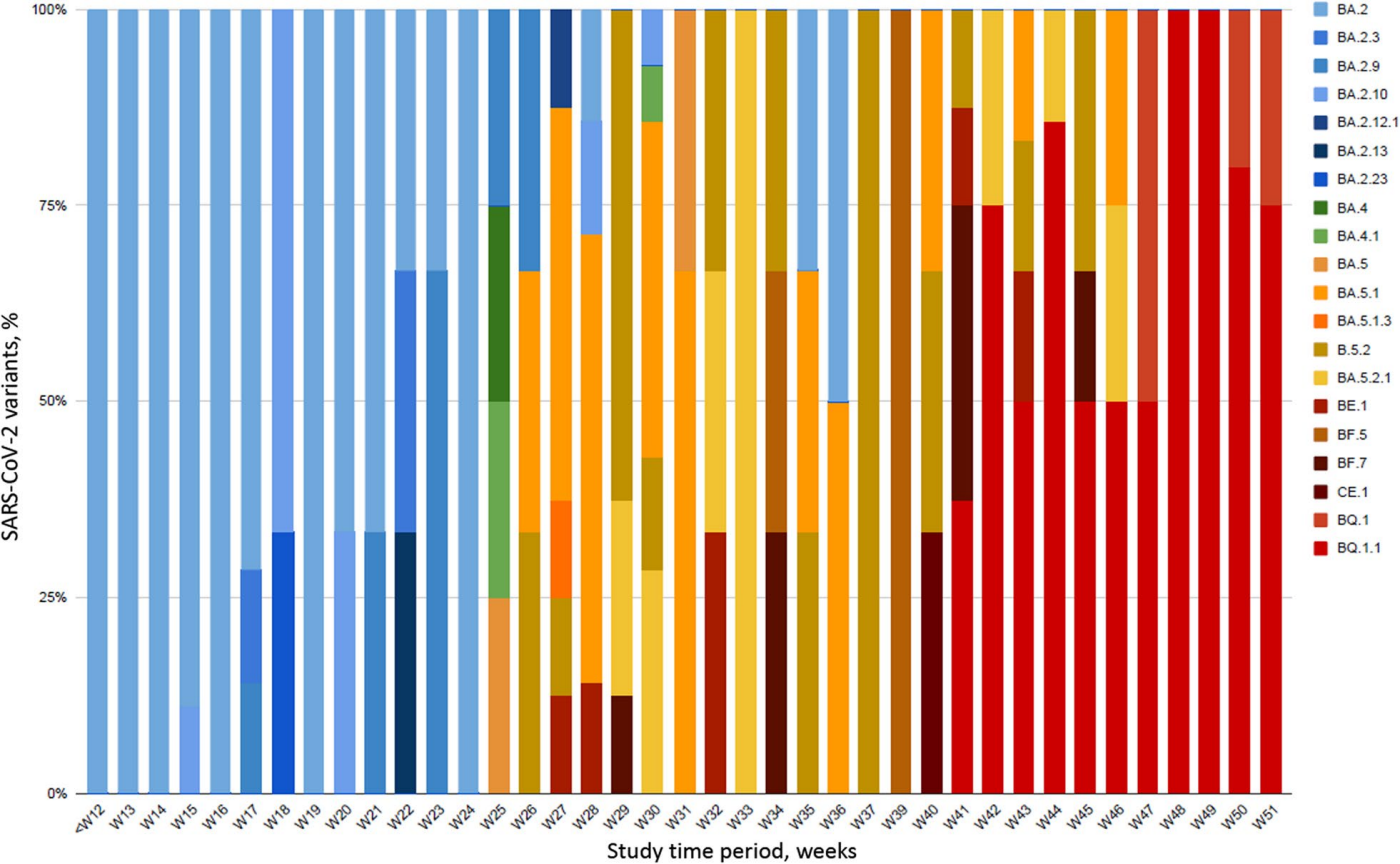
Dynamics of infecting SARS-CoV-2 variants during the 40-week study period. Patient-related samples (n = 158) corresponding to BA.2, BA.4, BA.5 and emerging sublineages are displayed over time. BA.2 sublineages are in blue, BA.4/BA.5 sublineages are in orange, brown, green and yellow and BQ.1.1 and other sublineages are in red, burgundy and black. The x-axis displays the study time period in weeks (w) from week 12, 2021 to week 51, 2022; The y-axis displays the percentage of each variant/sublineage at each time-point

Between December 7, 2021, and December 15, 2022, 486 patients were admitted in one of the 20 participating ICUs and included in the prospective SEVARVIR study. The current analysis comprised 158 patients infected with Omicron sublineages included between February 4, 2022, and December 15, 2022. They split into 3 groups: (i) BA.2 variants and their early sublineages (n = 50); (ii) early BA.4 and BA.5 sublineages (including BA.5.1 and BA.5.2) (n = 61); and (iii) recent emerging BA.5 sublineages (including BQ.1, BQ.1.1, BF.7, BE.1 and CE.1), assigned as the BQ.1.1 group (n = 47) (Figs. 1B and 2).

No statistically significant differences regarding age, gender and frequency of comorbidities were observed between patients infected with parental BA.2, BA.4/BA.5 and those infected with BQ.1.1 group sublineages, except more frequent obesity and less frequent immunosuppression in the latter group (Table 1). The proportion of patients who had received at least one dose of SARS-CoV-2 vaccine, as well as the median number of doses received, did not significantly differ between groups. However, the time elapsed since the third vaccine dose (for those who received it) and ICU admission was significantly longer for BQ.1.1 group-infected patients than for those from the two other groups of variants, including BA.2 and BA.4/BA.5 (307 days [190–508] vs 231 days [200–265] and 153 days [132–181], respectively; p = 0.013). The median time interval between the first symptoms and ICU admission was significantly shorter in the BQ.1.1 group than in early BA.4/BA.5 or BA.2-infected patients (3 [1–7] vs 4 [2–9] and 7 [3–13] days, respectively; p = 0.022). Other variables related to SARS-CoV-2 infection, including the median viral level in the upper respiratory tract, as measured by the cycle threshold in RT-PCR, and the prevalence of positive SARS-CoV-2 anti-S antibodies upon ICU admission, did not significantly differ between groups (Table 1).

**Table 1:** Clinical and biological characteristics of the 158 patients with severe SARS-CoV-2 infection at the time of their intensive care unit admission according to the infecting SARS-CoV-2 “sublineage groups” (BA.2 *vs* BA.4/BA.5 vs BQ.1.1 group)

	Data available	All patients	BA.2	BA.4/BA.5	BQ.1.1 group	p-value
		N = 158	N = 50	N = 61	N = 47	
Demographics and comorbidities						
Sex, females	158	50 (31.6)	13 (26.0)	19 (31.1)	18 (38.3)	0.426
Age, years	158	67.4 (± 13.9)	65.1 (± 13.2)	68.3 (± 10.8)	68.7 (± 14.1)	0.364
Diabetes	158	53 (34.4)	19 (38.0)	21 (36.2)	13 (28.3)	0.566
Obesity	158	41 (26.1)	8 (16.3)	15 (24.6)	18 (38.3)	**0.047**
Chronic heart failure	158	18 (11.7)	2 (4.0)	8 (13.8)	8 (17.4)	0.079
Hypertension	158	94 (61.0)	29 (58.0)	35 (60.3)	30 (65.2)	0.762
Chronic respiratory failure^a^	158	37 (24.2)	13 (26.0)	15 (25.9)	9 (20.0)	0.738
Chronic renal failure^b^	158	33 (21.4)	12 (24.0)	15 (25.9%)	6 (13.0)	0.247
Cirrhosis	158	3 (1.9)	1 (2.0)	2 (3.4)	0 (0)	0.777
Immunosuppression	158	63 (40.9)	28 (56.0)	23 (39.7)	12 (26.1)	**0.012**
Immunosuppression						
None		91 (59.1)	22 (44.0)	35 (60.3)	34 (73.9)	**< 0.0001**
Solid organ transplant		20 (13.0)	9 (18.0)	9 (15.5)	2 (4.3)	
Onco-hematological malignancies		20 (13.0)	7 (14.0)	7 (12.1)	6 (13.0)	
Others^c^		23 (14.9)	12 (24.0)	7 (12.1)	4 (8.7)	
Number of comorbidities^d^	158	2 (1;3)	2 (1;3)	2 (1;3)	2 (1;3)	0.694
Clinical frailty scale	156	3 (3;4)	4 (3;4)	3 (3;4)	3 (3;5)	0.751
SARS-CoV-2 infection and Vaccination						
Previous SARS-CoV-2 infection	158	8 (5.2)	3 (6.0)	4 (6.9)	1 (2.2)	0.598
SARS-CoV-2 vaccination	158	116 (75.8)	42 (84.0)	43 (74.1)	31 (68.9)	0.213
Number of doses among vaccinated	111	3 (3;3)	3 (3;3)	3 (3;3)	3 (3;4)	0.452
3rd dose- ICU admission^e^, days	15	190 (163;265)	153 (132;181)	231 (200;265)	307 (190;528)	**0.013**
Last dose—ICU admission^e^, days	25	182 (132;254)	153 (101;181)	200 (86;265)	257 (190;508)	0.157
SARS-CoV-2 serology at ICU admission						
Unavailable	158	80 (50.6)	18 (31.53%)	41 (67.2)	21 (44.7)	**0.008**
Negative^f^	158	22 (13.9)	8 (38.74%)	8 (13.1)	6 (12.8)	
Positive	158	56 (35.4)	24 (29.73%)	12 (19.7)	20 (42.6)	
First symptoms—ICU admission, days	158	5 (2;10)	7 (3;13)	4 (2;9)	3 (1;7)	**0.013**
SARS-CoV-2 RNA detection in nasopharyngeal swabs, Ct	114	20 (16;24)	21 (18;26)	20 (16;23)	19 (15;23)	0.123
Patients severity upon ICU admission and biological features						
WHO 10-point scale	157	6 (6;6)	6 (6;6)	6 (5;6)	6 (6;7)	0.598
SAPS II score	153	35 (28;45)	35 (30;43)	37 (30;47)	33 (27;46)	0.460
SOFA score	153	4 (3;7)	5 (3;7)	4 (3;7)	4 (3;7)	0.801
PaO_2_/FiO_2_ ratio, mmHg	154	150 (98;233)	158 (110;214)	150 (98;257)	140 (97;217)	0.856
ARDS criteria	154	110 (71.4)	37 (75.5)	41 (69.9)	62 (69.6)	0.791
Arterial lactate level, mM	154	1.6 (1.0;2.4)	1.5 (1.0;2.1)	1.5 (1.0;2.4)	1.8 (1.1;3.0)	0381
Blood leukocytes, G/L	156	9.9 (6.4;13.3)	9.8 (5.5;13.3)	8.6 (6.7;13.0)	10.2 (6.3;13.4)	0.187
Blood lymphocytes, G/L	124	0.5 (0.3;0.8)	0.5 (0.2;0.8)	0.4 (0.3;0.7)	0.6 (0.3;1.0)	0.491
Blood platelets, G/L	156	185 (133;262)	165 (111;249)	195 (140;266)	198 (135;278)	0.183
Serum urea level, mM	157	9 (6;15)	10 (6;15)	9 (6;18)	8 (6;13)	0.118
Serum creatinine level, µM	157	101 (71;164)	100 (71;154)	129 (76;222)	92 (69;137)	0.217
Bacterial coinfection	158	35 (22.3)	12 (24.0)	10 (16.7)	13 (27.7)	0.375
Thoracic CT-scan	157	87 (55.4)	30 (60.0)	29 (48.3)	28 (59.6)	0.373
Pulmonary embolism	156	5 (5.7)	1 (3.3)	2 (6.7)	2 (7.4)	0.685
Lung parenchyma involvement, %	55	40 (25;75)	75 (40;75)	35 (25;51)	40 (25;62)	0.063
Oxygen/ventilatory support						
Oxygen	158	35 (22.6)	7 (14.0)	16 (27.1)	12 (26.1)	0.584
High flow oxygen	158	62 (40.0)	25 (50.0)	22 (37.3)	15 (32.6)	
NIV/C-PAP	158	19 (12.3)	6 (12.0)	7 (11.9)	6 (13.0)	
Invasive MV	158	39 (25.2)	12 (24.0)	14 (23.7)	13 (28.3)	
ECMO support	158	1 (0.6)	1 (2.0)	0 (0)	0 (0)	0.618
Vasopressor support	158	28 (17.8)	8 (16.0)	10 (16.7)	10 (21.3)	0.780

Results are N(%), means (± standard deviation) or medians (interquartile range). ^a^requiring long-term oxygen treatment; ^b^defined as glomerular filtration rate < 60 mL/min/1.73 m^2 c^includes HIV infection, long-term corticosteroid treatment, and other immunosuppressive treatments; ^d^include diabetes, obesity, chronic heart, renal and respiratory failure, hypertension, cirrhosis, and immunosuppression; ^e^time lag between the last vaccination dose and ICU admission; ^f^defined as < 30 Binding Antibody Units (BAU)/mL; ARDS: acute respiratory distress syndrome; ICU: intensive care unit; Ct: cycle threshold; WHO: World Health Organization; SOFA: Sequential Organ Failure Assessment; SAPS II: Simplified Acute Physiology Score II; NIV: non-invasive ventilation; C-PAP; continuous-positive airway pressure; MV: mechanical ventilation; ECMO: extracorporeal mechanical ventilation; Two-tailed p-values come from unadjusted comparisons using Chi square or Fisher’s exact tests for categorical variables, and t-tests or Mann–Whitney tests for continuous variables, as appropriate. No adjustment for multiple comparisons was performed; Bolded p-values are significant at the p < 0.05 level

There was no significant difference between BA.2, BA.4/BA.5, and BQ.1.1 groups regarding the severity of the disease at ICU admission, as reflected by the SOFA and SAPS II scores and the WHO 10-point ordinal scale (Table 1). Invasive mechanical ventilation support was required in 25.2% (n = 39/158) of patients within 24 h of ICU admission, with no significant difference between groups. Only one BA.2-infected patient required extracorporeal membrane oxygenation (ECMO) support upon ICU admission, while none did in the two other sublineage groups.

During ICU stay, 31.2% (n = 49/158) of patients required invasive mechanical ventilation, with no significant differences between the three subvariant groups. There was also no significant difference between groups regarding the need for other organ supports, including vasopressors, renal replacement therapy or ECMO (Table 2). Only 2 patients (1.3%), not belonging to the BQ1.1 group, required ECMO support for refractory acute respiratory distress syndrome, contrasting with older studies reporting a need for ECMO support in 10 to 20% of patients infected with the ancestral, Alpha or Delta SARS-CoV-2 variants [9, 25, 26]. Patients from the BQ.1.1 group had a significantly shorter duration of ICU stay than those from the two other groups (6 days [4–11] vs 7 days [3–15] and 11 days [5–22] days, respectively; p = 0.038). Day-28 mortality was not significantly different between the three groups. There were no significant between-group differences of COVID-19 management, with 72.0% (n = 113/157) of patients who received dexamethasone and 19.4% (n = 30/155) tocilizumab. Monoclonal antibodies were used in 13% (n = 18/158) of patients and nirmatrelvir-ritonavir in 8.9% (n = 14/157).

**Table 2:** Intensive care management and outcomes of patients with severe SARS-CoV-2 infection (n = 158) during their intensive care unit stay according to the SARS-CoV-2 infecting “sublineage groups” (BA.2 *vs* BA.4/BA.5 vs BQ.1.1 group)

	Data available	All patients	BA.2	BA.4/BA.5	BQ.1.1 group	p-value
		N = 158	N = 50	N = 61	N = 47	
Invasive MV	158	49 (31.2)	16 (32.0)	20 (33.3)	13 (37.7)	0.812
Prone positioning	158	28 (19.7)	11 (23.9)	11 (20.0)	6 (14.6)	0.553
MV duration, days	158	9 (3;18)	12 (5;25)	9 (2;17)	4 (2;14)	0.213
Live-ventilator free days at day 28	158	28 (0;28)	28 (0;28)	28 (0;28)	28 (6;28)	0.823
ECMO support	158	2 (1.3)	1 (2.0)	1 (1.7)	0 (0)	1.000
Vasopressor support	158	46 (29.5)	13 (26.0)	19 (31.7)	14 (30.4)	0.797
Renal replacement therapy	158	16 (10.2)	4 (8.0)	7 (11.7)	5 (10.6)	0.847
Ventilator-acquired pneumonia (among IMV)^a^	49	20 (40.8)	7 (43.8)	8 (40.0)	5 (38.5)	1.000
CAPA	158	8 (5.1)	5 (10.0)	2 (3.3)	1 (2.2)	0.160
Dexamethasone	158	113 (72.0)	38 (76.0)	42 (70.0)	33 (70.2)	0.773
Other steroids	158	0	0	0	0	–
Tocilizumab	158	30 (19.4)	14 (28.0)	11 (18.6)	5 (10.9)	0.104
Nirmatrelvir-ritonavir	157	14 (8.9)	2 (4.0)	10 (16.7)	2 (4.3)	**0.028**
Monoclonal antibodies	158	18 (13.0)	10 (20.8)	2 (3.8)	6 (15.8)	**0.022**
Casirivimab-Imdevimab	158	1 (5.6)	0 (0)	0 (0)	6 (15.8)	0.444
Tixagevimab-Cilgavimab	158	17 (94.4)	10 (100.0)	2 (100.0)	5 (3.3)	0.444
Sotrovimab	158	0	0	0	0	–
Duration of ICU stay, days	158					
All patients	158	8 (4; 16)	11 (5; 22)	7 (3; 15)	6 (4; 12)	**0.048**
Survivors only	121	7 (3; 16)	9 (5; 22)	6 (3; 14)	6 (4; 15)	0.069
Day-28 mortality	158	37 (23.7)	11 (22.0)	16 (26.7)	10 (21.7)	0.791

Results are N (%), means (± standard deviation) or medians (interquartile range); MV: mechanical ventilation; ECMO: extracorporeal mechanical ventilation; VAP: ventilator-acquired pneumonia; IMV: invasive mechanical ventilation; CAPA: COVID-19-associated pulmonary aspergillosis; ^a^VAP episodes were recorded per definition in patients under IMV since more than 48 h; Two-tailed p-values come from unadjusted comparisons using Chi square or Fisher’s exact tests for categorical variables, and t-tests or Mann–Whitney tests for continuous variables, as appropriate. No adjustment for multiple comparisons was performed; Bolded p-values are significant at the p < 0.05 level

A sensitivity analysis comparing patients with BQ.1.1 infection with the two other groups after excluding other emerging sublineages (n = 34) showed similar results (Additional file 1: Tables S2 and S3).

As expected, patients dead at day 28 were older and had more severe disease than survivors. They also more frequently had onco-hematological diseases (Additional file 1: Table S4). We found no significant association between Omicron sublineages and day 28 mortality in uni- and multivariable mortality adjusting on age, SOFA and immunosuppression (Table 3).

**Table 3:** Multivariable logistic regression analysis of factors associated with day 28 mortality

	Alive N = 119	Dead N = 37		
	N	N	OR (CI 95%)	p value
Age, years	119	37	1.04 (1.01; 1.08)	**0.012**
SOFA score	116	36	1.16 (1.03; 1.30)	**0.017**

	N (H%)	N (H%)	OR (CI 95%)	p value
Subvariant lineage				
BA.2	39 (78.0%)	11 (22.0%)	1 (ref.)	0.791
BA.4/5	44 (73.3%)	16 (26.7%)	1.29 (0.53; 3.11)	0.572
BQ.1.1 group	36 (78.3%)	10 (21.7%)	0.98 (0.37; 2.59)	0.975
Gender, female	38 (79.2%)	10 (20.8%)	0.79 (0.35;1.80)	0.573
Immunosuppression	43 (68.2%)	20 (31.7%)	2.00 (0.95; 4.22)	0.070

aOR: adjusted odds ratio; CI: confidence interval; bolded values are significant at the p < 0.05 level

**Fig. 3:**
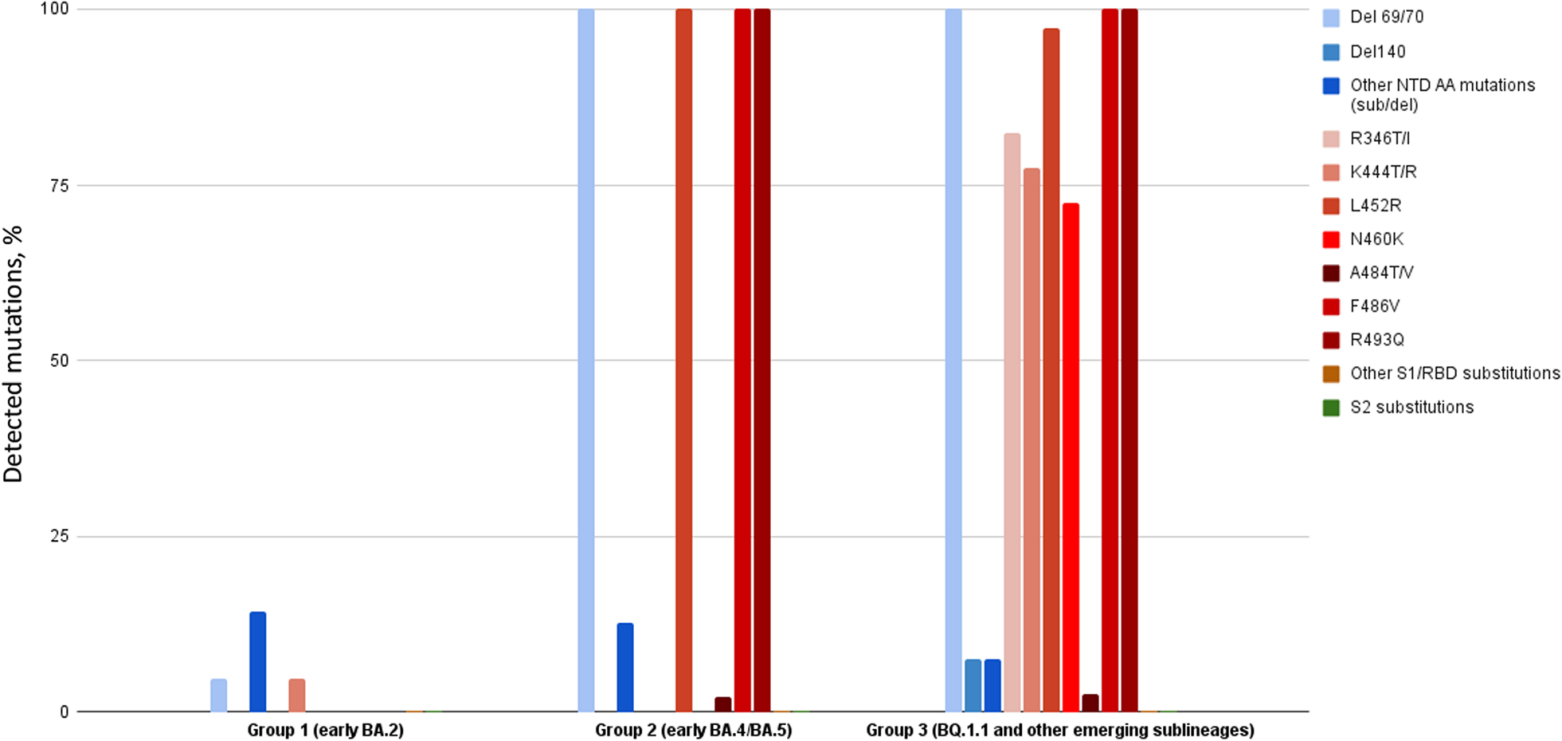
Prevalence of amino acid substitutions and deletions in Spike RBD in BA.2, BA.4/BA.5, and BQ.1.1-related group viral sequences; The percentage of detected mutations (amino acid substitutions and deletions) per group is displayed on the y-axis, relative to the original Omicron BA.2 reference sequence (SARS-CoV-2/human/USA/FL-CDC-STM-77CPCCUR3/2022). Other individual NTD (N-terminal domain) mutations include H49Y, W64R, M153I, M177L, I197T, Del211, L212I, A222S, T250I, P251H, V289I and S316F; other S1/RBD mutations include N354K, I358L, T376S, T547K, T572I and N568S; other S2 mutations include T547K, T572I, H625R, A642G, A647V, E654K, N658S, N856S, V963F, A1020S, T1117I, P1143L, E1144Q and S1249P

High-coverage full-length viral genome sequence analysis (> 90% full-length genome and > 95% full-length spike gene) was obtained in 140 of the 158 patients. A higher number of key amino acid substitutions, which confer further immune escape ability over pre-existing lineages, was found in the RBD of the BQ.1.1 group of patients than in those from the BA.4/BA.5 and BA.2 groups. Additional substitutions were detected in S2 in the three groups (Fig. 3). No significant association was found between any SARS-CoV-2 substitution and/or deletion on the one hand and survival on the other hand over hospital follow-up (Table 4).

**Table 4:** Relationship between major amino-acid substitutions in spike receptor binding domain (RBD) and hospital mortality in univariable Cox proportional hazard regression analysis

Non synonymous mutations in Spike RBD	N^a^	HR 95% CI	P value
R346T/I	34	1.10 [0.47; 2.57]	0.821
K444T/R	29	1.29 [0.62; 3.38]	0.389
L452R	81	1.33 [0.49; 3.56]	0.575
N460K	28	1.49 [0.64; 3.48]	0.353
F486V	81	1.33 [0.49; 3.56]	0.575

HR 95% CI: Hazard ratio 95% Confidence Interval; ^a^Number of patients with the mutation on a total of 108 patient-related samples for which full-length viral genome sequence analysis yielding high coverage with > 95% on spike gene was performed

## Discussion

This study is the first describing the clinical phenotype associated with new emerging Omicron sublineages (e.g., BF.7 and BQ.1.1) in patients with severe COVID-19 requiring hospitalization in the ICU. Our data provide reassuring evidence that these emerging sublineages do not cause more severe outcomes than BA.2 and BA.5 variants that emerged and spread earlier in the population. We observed unexpected phenotype differences comparing Omicron sublineages, with more frequent obesity, less frequent immunosuppression and shorter duration of ICU stay in patients infected with BQ.1.1, as compared to those infected with other sublineages.

No significant difference was observed between Omicron sublineage groups regarding the severity of the disease at ICU admission, the need for organ failure support during ICU stay, or day 28 mortality. Notably, the emerging sublineages did not cause severe ARDS requiring ECMO support, in contrast with what has been observed during pre-Omicron COVID-19 waves [9, 25, 26]. In fact, with the emergence of new Omicron sublineages, the clinical phenotype of COVID-19-associated acute respiratory failure seems to be evolving towards less severe disease, as attested by only one third of patients of the current cohort who required invasive mechanical ventilation support, compared to almost 50% of Omicron BA.1-infected patients, 57% of Delta-infected patients [9] and 63% of patients infected with the Wuhan variant [25]. While immunosuppression remained associated with a high mortality, as previously shown with BA.1 [9], the high rate of BQ1.1-infected patients with cardiorespiratory comorbidities, together with a non-significant decrease in CT-scan lung parenchyma involvement, and the shorter ICU stay, as compared with previous series of critically ill COVID-19 patients [9, 26], suggests that a significant subset of patients presented with SARS-CoV-2-associated decompensated heart failure or acute exacerbation of chronic respiratory failure rather than true SARS-CoV-2-associated pneumonia.

Patients infected with sublineage BQ.1.1 had more frequent obesity and were less frequently immunosuppressed than those infected with other Omicron sublineages in our study. Such finding was unexpected since immunosuppression has been the most frequently associated comorbidity in COVID-19 patients infected with the Omicron variant, since the BA.1 “ancestral” Omicron sublineage [9, 27], reported in almost 50% of cases. On the other hand, the higher prevalence of obesity, a previously reported risk factor of severity with previous SARS-CoV-2 variants, including the ancestral variant, is consistent with previous data reporting obesity as a risk factor of severity [28]. The re-emergence of a patient population with no immunosuppression and more classical comorbidities such as obesity, as encountered in pre-Omicron surges, might be consistent with a decrease in post-vaccinal protection, as suggested by a greater delay between the 3rd vaccinal dose and ICU admission in BQ.1.1 patients than in others in our cohort, and data showing a lower neutralization activity in patients infected with BQ.1.1 than with BA.5 or BA.1 subvariants, even after bivalent booster vaccination [29]. The median delay between the first symptoms and ICU admission was significantly shorter in the BQ.1.1 group, possibly accounting for the greater ability of these sublineages to increase cell–cell fusion compared to their parental variants [30].

Overall, the severity of the disease in patients hospitalized in the ICU and their day-28 mortality did not differ across different Omicron sublineage groups. However, the reported increased transmissibility and antibody neutralization escape capacity of sublineage BQ.1.1 [12] make the use of monoclonal antibodies inefficacious in this population [10], including Bebtelovimab, while Sotrovimab might remain weakly active [13]. In contrast, direct-acting antiviral agents, such as paxlovid, are active on new emerging sublineages [31]. The 20% mortality rate observed in our cohort of critically ill patients with COVID-19 corroborates the recent update of the World Health Organization living guideline on therapeutics and COVID-19 (https://www.who.int/publications/i/item/WHO-2019-nCoV-therapeutics-2022.4), which recommends the early use of nirmatrelvir-ritonavir (i.e., within the first five days of disease onset) in patients with non-severe COVID-19 at highest risk of hospitalization. This is particularly true in patients with lower exposure to vaccination, in whom the severity of infection is expected to be greater, as suggested by the COVID-19 surge during winter 2022–2023 in China, mainly driven by the BF.7 Omicron sublineage.

Our study has limitations, including a limited sample size in the BQ.1.1 group, limiting our statistical power to perform subgroup analyses and adjust for confounding variables. Indeed, our current multivariable analysis assessing the relationship between Omicron sublineages and day-28 mortality adjusted for key confounders such as age, gender, SOFA score and immunosuppression status, but the relatively low number of events (n = 36 deaths) precluded inflating the number of independent variables. Still, other confounders might have been relevant to include in the model and have been previously shown to be associated with mortality (e.g., diabetes, obesity, chronic respiratory and cardiac diseases [30], vaccination status). In this study, we did not define a priori the sample size of the study, making our study findings exploratory. This is because we aimed at capturing the dynamics of emerging SARS-CoV-2 sublineages and analyzing their phenotype and relationship with mortality in real time. The overall patient recruitment, in spite of a high number of participating centers (n = 20), reflects a lower epidemic activity during the study period than during previous COVID-19 waves. Moreover, we did not explore in depth the physiological mechanisms of acute respiratory failure (i.e., collecting hemodynamic and respiratory physiological data), precluding firm conclusions to be made regarding the contribution of SARS-CoV-2 pneumonia versus that of decompensated underlying cardiopulmonary comorbidities. In-depth mutation analysis could not be performed for all patients, because full-length viral genome sequences were analyzed only when the sequencing coverage was greater than 90% (> 95% in the Spike gene). However, our study also has major strengths, in particular the constitution of a unique national prospective multicenter cohort of well-phenotyped critically ill patients and the availability of full-length SARS-CoV-2 genome sequences for the vast majority of them, allowing for prospective exploration of the clinical consequences of all waves of infection related to emerging and spreading SARS-CoV-2 variants.

In conclusion, critically-ill patients with Omicron BQ.1.1 infection showed a different clinical phenotype than other patients infected with earlier Omicron sublineage. However, there was no significant difference between Omicron sublineage groups regarding the severity of the disease at ICU admission, need for organ failure support during ICU stay, nor day 28 mortality.

## Supplementary Information

**Additional file 1: Table S1.** List of centres participating in the SEVARVIR study. **Table S2.** Clinical and biological characteristics of the 145 patients with severe SARS-CoV-2 infection at the time of their intensive care unit admission according to the infecting SARS-CoV-2 “sublineage groups” (BA.2 *vs* BA.4/BA.5 vs BQ.1.1). **Table S3.** Intensive care management and outcomes of patients with severe SARS-CoV-2 infection (n = 145) during their intensive care unit stay according to the SARS-CoV-2 infecting “sublineage groups” (BA.2 *vs* BA.4/BA.5 vs BQ.1.1). **Table S4.** Clinical and biological characteristics of the 145 patients with severe SARS-CoV-2 infection at the time of their intensive care unit admission according to their vital status at day 28.

## Data Availability

All the datasets generated during and/or analyzed during the current study are available from the corresponding author on reasonable request (S.F.).

## Abbreviations

ARDS: Acute respiratory distress syndrome
Ct: Cycle threshold
ECMO: Extracorporeal membrane oxygenation
ICU: Intensive care unit
RBD: Receptor-binding domain
RT-PCR: Reverse transcriptase-polymerase chain reaction
SAPS: Simplified acute physiology score
SD: Standard deviation
SOFA: Sequential organ failure assessment

## References

[1] Varghese R, Kumar D, Sharma R (2023). Global threat from novel SARS-CoV-2 variants, BF.7, XBB.1.5, BQ.1, and BQ.1.1: variants of concern?. Hum Cell.

[2] Novazzi F, Giombini E, Rueca M, Baj A, Fabeni L, Genoni A (2023). Genomic surveillance of SARS-CoV-2 positive passengers on flights from China to Italy, December 2022. Euro Surveill.

[3] Mykytyn AZ, Rosu ME, Kok A, Rissmann M, Amerongen G van, Geurtsvankessel C, et al. Antigenic mapping of emerging SARS-CoV-2 omicron variants BM.1.1.1, BQ.1.1, and XBB.1. Lancet Microbe [Internet]. 2023;0. Available from: https://www.thelancet.com/journals/lanmic/article/PIIS2666-5247(22)00384-6/fulltext. 10.1016/S2666-5247(22)00384-6 PMC9842387 36657480

[4] Wang Q, Iketani S, Li Z, Liu L, Guo Y, Huang Y (2023). Alarming antibody evasion properties of rising SARS-CoV-2 BQ and XBB subvariants. Cell.

[5] DP-EFFECT-BRAZIL investigators. Variants of concern and clinical outcomes in critically ill COVID-19 patients. Intensive Care Med. 2023;1–3. 10.1007/s00134-023-07039-2 PMC10108805 37067557

[6] Saito A, Tamura T, Zahradnik J, Deguchi S, Tabata K, Anraku Y (2022). Virological characteristics of the SARS-CoV-2 Omicron BA.2.75 variant. Cell Host Microbe.

[7] Kimura I, Yamasoba D, Tamura T, Nao N, Suzuki T, Oda Y (2022). Virological characteristics of the SARS-CoV-2 Omicron BA.2 subvariants, including BA.4 and BA.5. Cell.

[8] Ito J, Suzuki R, Uriu K, Itakura Y, Zahradnik J, Kimura KT (2023). Convergent evolution of SARS-CoV-2 Omicron subvariants leading to the emergence of BQ.1.1 variant. Nat Commun.

[9] de Prost N, Audureau E, Heming N, Gault E, Pham T, Chaghouri A (2022). Clinical phenotypes and outcomes associated with SARS-CoV-2 variant Omicron in critically ill French patients with COVID-19. Nat Commun.

[10] Cox M, Peacock TP, Harvey WT, Hughes J, Wright DW, Willett BJ (2023). SARS-CoV-2 variant evasion of monoclonal antibodies based on in vitro studies. Nat Rev Microbiol.

[11] RECOVERY Collaborative Group (2022). Casirivimab and imdevimab in patients admitted to hospital with COVID-19 (RECOVERY): a randomised, controlled, open-label, platform trial. Lancet.

[12] Miller J, Hachmann NP, Collier AY, Lasrado N, Mazurek CR, Patio RC (2023). Substantial neutralization escape by SARS-CoV-2 Omicron Variants BQ.1.1 and XBB.1. N Engl J Med.

[13] Planas D, Bruel T, Staropoli I, Guivel-Benhassine F, Porrot F, Maes P (2023). Resistance of Omicron subvariants BA.2.75.2, BA.4.6, and BQ.1.1 to neutralizing antibodies. Nat Commun.

[14] Rockwood K, Song X, MacKnight C, Bergman H, Hogan DB, McDowell I (2005). A global clinical measure of fitness and frailty in elderly people. CMAJ.

[15] WHO Working Group on the Clinical Characterisation and Management of COVID-19 infection (2020). A minimal common outcome measure set for COVID-19 clinical research. Lancet Infect Dis.

[16] Vincent JL, Moreno R, Takala J, Willatts S, De Mendonça A, Bruining H (1996). The SOFA (Sepsis-related Organ Failure Assessment) score to describe organ dysfunction/failure. On behalf of the Working Group on Sepsis-Related Problems of the European Society of Intensive Care Medicine. Intensive Care Med.

[17] Le Gall JR, Lemeshow S, Saulnier F (1993). A new Simplified Acute Physiology Score (SAPS II) based on a European/North American multicenter study. JAMA.

[18] Ranieri VM, Rubenfeld GD, Thompson BT, Ferguson ND, Caldwell E, ARDS Definition Task Force (2012). Acute respiratory distress syndrome: the Berlin Definition. JAMA.

[19] Leone M, Bouadma L, Bouhemad B, Brissaud O, Dauger S, Gibot S (2018). Brief summary of French guidelines for the prevention, diagnosis and treatment of hospital-acquired pneumonia in ICU. Ann Intensive Care.

[20] Koehler P, Bassetti M, Chakrabarti A, Chen SCA, Colombo AL, Hoenigl M (2021). Defining and managing COVID-19-associated pulmonary aspergillosis: the 2020 ECMM/ISHAM consensus criteria for research and clinical guidance. Lancet Infect Dis.

[21] Bhoyar RC, Jain A, Sehgal P, Divakar MK, Sharma D, Imran M (2021). High throughput detection and genetic epidemiology of SARS-CoV-2 using COVIDSeq next-generation sequencing. PLoS ONE.

[22] Rambaut A, Holmes EC, O’Toole Á, Hill V, McCrone JT, Ruis C (2020). A dynamic nomenclature proposal for SARS-CoV-2 lineages to assist genomic epidemiology. Nat Microbiol.

[23] Ma KC, Shirk P, Lambrou AS, Hassell N, Zheng X-Y, Payne AB (2023). Genomic Surveillance for SARS-CoV-2 Variants: circulation of Omicron Lineages—United States, January 2022-May 2023. MMWR Morb Mortal Wkly Rep.

[24] La Rosa G, Brandtner D, Bonanno Ferraro G, Veneri C, Mancini P, Iaconelli M (2023). Wastewater surveillance of SARS-CoV-2 variants in October-November 2022 in Italy: detection of XBB.1, BA.2.75 and rapid spread of the BQ.1 lineage. Sci Total Environ.

[25] COVID-ICU Group on behalf of the REVA Network and the COVID-ICU Investigators (2021). Clinical characteristics and day-90 outcomes of 4244 critically ill adults with COVID-19: a prospective cohort study. Intensive Care Med.

[26] Fourati S, Audureau E, Arrestier R, Marot S, Dubois C, Voiriot G (2022). SARS-CoV-2 genomic characteristics and clinical impact of SARS-CoV-2 viral diversity in critically ill COVID-19 patients: a prospective multicenter cohort study. Viruses.

[27] Vieillard-Baron A, Flicoteaux R, Salmona M, Chariot A, De Maupeou D’Ableiges B, Darmon M (2022). Omicron variant in the critical care units of Paris metropolitan area the reality research group. Am J Respir Crit Care Med.

[28] Williamson EJ, Walker AJ, Bhaskaran K, Bacon S, Bates C, Morton CE (2020). Factors associated with COVID-19-related death using OpenSAFELY. Nature.

[29] Davis-Gardner ME, Lai L, Wali B, Samaha H, Solis D, Lee M (2023). Neutralization against BA.2.75.2, BQ.1.1, and XBB from mRNA Bivalent Booster. N Engl J Med.

[30] Qu P, Evans JP, Faraone J, Zheng Y-M, Carlin C, Anghelina M (2022). Distinct neutralizing antibody escape of SARS-CoV-2 Omicron Subvariants BQ.1, BQ.1.1, BA.4.6, BF.7 and BA.2.75.2 [Internet]. bioRxiv.

[31] Cho J, Shin Y, Yang J-S, Kim JW, Kim K-C, Lee J-Y (2023). Evaluation of antiviral drugs against newly emerged SARS-CoV-2 Omicron subvariants. Antiviral Res.

