# Supplementary material for: Clinical phenotypes and outcomes associated with SARS-CoV-2 Omicron variants BA.2, BA.5 and BQ.1.1 in critically ill patients with COVID-19: a prospective, multicenter cohort study
Source: Intensive Care Med Exp. 2023 Aug 7;11:48. doi: 10.1186/s40635-023-00536-0 (PMC10404579; doi:10.1186/s40635-023-00536-0)
Supplement: Supplementary file 1 — Additional file 1: Table S1. List of centres participating in the SEVARVIR study. Table S2. Clinical and biological characteristics of the 145 patients with severe SARS-CoV-2 infection at the time of their intensive care unit admission according to the infecting SARS-CoV-2 “sublineage groups” (BA.2 vs BA.4/BA.5 vs BQ.1.1). Table S3. Intensive care management and outcomes of patients with severe SARS-CoV-2 infection (n = 145) during their intensive care unit stay according to the SARS-CoV-2 infecting “sublineage groups” (BA.2 vs BA.4/BA.5 vs BQ.1.1). Table S4. Clinical and biological characteristics of the 145 patients with severe SARS-CoV-2 infection at the time of their intensive care unit admission according to their vital status at day 28. [file 40635_2023_536_MOESM1_ESM.docx]

**Clinical phenotypes and outcomes associated with SARS-CoV-2 Omicron variants BA.2, BA.5 and BQ.1.1 in critically ill patients with COVID-19: a prospective, multicenter cohort study**

**Additional document**

**Content**

Additional Table S1………………………………………………………………….Page 2

Additional Table S2………………………………………………………………….Page 3

Additional Table S3………………………………………………………………….Page 6

Additional Table S4………………………………………………………………….Page 7

Additional **Table S1. List of centres participating in the SEVARVIR study**

| **n°** | **Centre** | **Department** |
| --- | --- | --- |
| 001 | Henri Mondor, Créteil | Medical and Surgical ICUs |
| 002 | Cochin, Paris | Medical ICU |
| 003 | Saint-Louis, Paris | Medical ICU |
| 004 | Pitié-Salpêtrière, Paris | Medical ICU |
| 005 | Saint-Antoine, Paris | Medical ICU |
| 006 | Pitié-Salpêtrière, Paris | Pulmonary and Medical ICU |
| 007 | Bichat, Paris | Medical ICU |
| 008 | Tenon, Paris | ICU |
| 009 | Avicenne, Bobigny | ICU |
| 010 | Louis Mourier, Colombes | ICU |
| 011 | Bicêtre, Le Kremlin-Bicêtre | Medical ICU |
| 012 | Raymond Poincaré, Garches | Medical ICU |
| 013 | Ambroise-Paré,  Boulogne-Billancourt | ICU |
| 014 | Marc Jacquet, Melun | ICU |
| 015 | CH Sud Francilien, Jossigny | ICU |
| 016 | CH Victor Dupouy, Argenteuil | ICU |
| 017 | Sainte Camille,  Bry sur Marne | ICU |
| 018 | CHU de Strasbourg | ICU |
| 019 | CHU de Lille | ICU |
| 020 | CHRU de Nancy,  Hôpitaux de Brabois - Nancy | Medical ICU |

Additional **Table S2.** Clinical and biological characteristics of the 145 patients with severe SARS-CoV-2 infection at the time of their intensive care unit admission according to the infecting SARS-CoV-2 “sublineage groups” (BA.2 *vs* BA.4/BA.5 vs BQ.1.1).

|  | | **Available data** | **All patients** | **BA.2** | **BA.4/BA.5** | **BQ.1.1** | **p-value** |
| --- | --- | --- | --- | --- | --- | --- | --- |
|  |  |  | N=145 | N=50 | N=61 | N=34 |  |
| ***Demographics and comorbidities*** | |  |  |  |  |  |  |
| Sex, females |  | 145 | 46 (31.7) | 13 (26.0) | 19 (31.1) | 14 (41.2) | 0.338 |
| Age, years |  | 145 | 67.1 (±14.0) | 65.1 (±13.2) | 68.3 (±10.8) | 67.8 (±14.7) | 0.461 |
| Diabetes |  | 145 | 52 (36.9) | 19 (38.0) | 21 (36.2) | 12 (36.4) | 0.979 |
| Obesity |  | 145 | 38 (26.4) | 8 (16.3) | 15 (24.6) | 15 (44.1) | **0.017** |
| Chronic heart failure |  | 145 | 15 (11.7) | 2 (4.0) | 8 (13.8) | 5 (15.2) | 0.146 |
| Hypertension |  | 145 | 87 (61.7) | 29 (58.0) | 35 (60.3) | 23 (69.7) | 0.541 |
| Chronic respiratory failure |  | 145 | 33 (23.6) | 13 (26.0) | 15 (25.9) | 5 (15.6) | 0.528 |
| Chronic renal failure |  | 145 | 32 (22.7) | 12 (24.0) | 15 (25.9) | 5 (15.2) | 0.523 |
| Cirrhosis |  | 145 | 3 (2.1) | 1 (2.0) | 2 (3.4) | 0 (0) | 0.791 |
| Immunosuppression |  | 145 | 60 (42.6) | 28 (56.0) | 23 (39.7) | 9 (27.3) | **0.029** |
| Immunosuppression | None | 145 | 81 (57.4) | 22 (44.0) | 35 (60.3) | 24 (73.9) | 0.183 |
|  | Solid organ transplant | 145 | 20 (14.2) | 9 (18.0) | 9 (15.5) | 2 (4.3) |  |
|  | Onco-hematological malignancies | 145 | 18 (12.8) | 7 (14.0) | 7 (12.1) | 4 (13.0) |  |
|  | Others^a^ | 145 | 22 (15.6) | 12 (24.0) | 7 (12.1) | 3 (8.7) |  |
| Number of comorbidities |  | 145 | 2 (1;3) | 2 (1;3) | 2 (1;3) | 2 (1;3) | 0.972 |
| Clinical frailty scale |  | 143 | 3 (3;4) | 4 (3;4) | 3 (3;4) | 4 (3;5) | 0.724 |
| ***SARS-CoV-2 infection and Vaccination*** | |  |  |  |  |  |  |
| Previous SARS-CoV-2 infection | | 145 | 7 (5.0) | 3 (6.0) | 4 (6.9) | 0 (0.0) | 0.379 |
| SARS-CoV-2 vaccination | | 145 | 109 (77.9) | 42 (84.0) | 43 (74.1) | 24 (75.0) | 0.425 |
| Number of doses among vaccinated | | 104 | 3 (3;3) | 3 (3;3) | 3 (3;3) | 3 (3;4) | 0.574 |
| 3^rd^ dose- ICU admission^b^, days | | 14 | 191 (163;265) | 153 (132;181) | 231 (200;265) | 417 (307;528) | **0.012** |
| Last dose - ICU admission^b^, days | | 23 | 182 (132;265) | 153 (101;181) | 200 (86;265) | 407 (257;518) | **0.024** |
| SARS-CoV-2 serology at ICU admission | Unavailable | 145 | 75 (51.7) | 18 (31.53%) | 41 (67.2) | 16 (44.1) | **0.007** |
|  | Negative^c^ | 145 | 19 (13.1) | 8 (38.74%) | 8 (13.1) | 3 (8.8) |  |
|  | Positive | 145 | 51 (35.2) | 24 (29.73%) | 12 (19.7) | 15 (44.1) |  |
| First symptoms - ICU admission, days | | 145 | 5 (2;10) | 7 (3;13) | 4 (2;9) | 2 (1;7) | **0.017** |
| SARS-CoV-2 RNA detection in nasopharyngeal swabs, Ct | | 103 | 20 (16;24) | 21 (18;26) | 20 (16;23) | 19 (15;23) | 0.141 |
| ***Patients severity upon ICU admission and biological features*** | |  |  |  |  |  |  |
| WHO 10-point scale |  | 144 | 6 (6;6) | 6 (6;6) | 6 (5;6) | 6 (5;7) | 0.594 |
| SAPS II score |  | 141 | 35 (29;44) | 35 (30;43) | 37 (30;47) | 33 (26;45) | 0.407 |
| SOFA score |  | 141 | 4 (3;7) | 5 (3;7) | 4 (3;7) | 4 (3;6) | 0.801 |
| PaO_2_/FiO_2_ ratio, mmHg |  | 142 | 153 (98;237) | 158 (110;214) | 150 (98;257) | 144 (98;253) | 0.996 |
| Arterial lactate level, mM |  | 141 | 1.7 (1.0;2.4) | 1.5 (1.0;2.1) | 1.5 (1.0;2.4) | 1.9 (1.2;3.0) | 0349 |
| Blood leukocytes, G/L |  | 143 | 10.0 (6.5;13.6) | 9.8 (5.5;13.3) | 8.6 (6.7;13.0) | 10.6 (6.5;14.0) | 0.187 |
| Blood lymphocytes, G/L |  | 115 | 0.5 (0.3;0.9) | 0.5 (0.2;0.8) | 0.4 (0.3;0.7) | 0.6 (0.4;1.0) | 0.354 |
| Blood platelets, G/L |  | 143 | 186 (131;266) | 165 (111;249) | 195 (140;266) | 208 (135;305) | 0.125 |
| Serum urea level, mM |  | 144 | 9 (6;15) | 10 (6;15) | 9 (6;18) | 8 (5;12) | 0.475 |
| Serum creatinine level, µM |  | 144 | 104 (73;171) | 100 (71;154) | 129 (76;222) | 92 (71;150) | 0.146 |
| Bacterial coinfection |  | 145 | 31 (21.5) | 12 (24.0) | 10 (16.7) | 9 (26.5) | 0.470 |
| Oxygen/ventilatory support | Oxygen | 145 | 35 (24.5) | 7 (14.0) | 16 (27.1) | 12 (35.3) | 0.265 |
|  | High flow oxygen | 145 | 57 (39.9) | 25 (50.0) | 22 (37.3) | 10 (29.4) |  |
|  | NIV/C-PAP | 145 | 15 (10.5) | 6 (12.0) | 7 (11.9) | 2 (5.9) |  |
|  | Invasive MV | 145 | 36 (25.2) | 12 (24.0) | 14 (23.7) | 10 (29.4) |  |
| ECMO |  | 145 | 1 (0.7) | 1 (2.0) | 0 (0) | 0 (0) | 0.583 |
| Vasopressor support |  | 145 | 24 (16.7) | 8 (16.0) | 10 (16.7) | 6 (17.6) | 0.980 |

Results are N(%), means (±standard deviation) or medians (interquartile range). ^a^includes HIV infection, long-term corticosteroid treatment, and other immunosuppressive treatments; ^b^time lag between the last vaccination dose and ICU admission; ^c^defined as < 30 Binding Antibody Units (BAU)/mL; ICU: intensive care unit; Ct: cycle threshold; WHO: World Health Organization; SOFA: Sequential Organ Failure Assessment; SAPS II: Simplified Acute Physiology Score II; NIV: non-invasive ventilation; C-PAP; continuous-positive airway pressure; MV: mechanical ventilation; ECMO: extracorporeal mechanical ventilation; Two-tailed p-values come from unadjusted comparisons using Chi square or Fisher’s exact tests for categorical variables, and t-tests or Mann-Whitney tests for continuous variables, as appropriate. No adjustment for multiple comparisons was performed; **Bolded** p-values are significant at the p<0.05 level.

Additional **Table S3.** Intensive care management and outcomes of patients with severe SARS-CoV-2 infection (n=145) during their intensive care unit stay according to the SARS-CoV-2 infecting “sublineage groups” (BA.2 *vs* BA.4/BA.5 vs BQ.1.1).

|  |  | **Available data** | **All patients** | **BA.2** | **BA.4/BA.5** | **BQ.1.1** | **p-value** |
| --- | --- | --- | --- | --- | --- | --- | --- |
|  |  |  | N=145 | N=50 | N=61 | N=34 |  |
| Invasive MV |  | 145 | 46 (31.9) | 16 (32.0) | 20 (33.3) | 10 (29.4) | 0.926 |
| Prone positioning |  | 145 | 25 (18.8) | 11 (23.9) | 11 (20.0) | 3 (9.4) | 0.259 |
| MV duration, days |  | 145 | 9 (2;18) | 12 (5;25) | 9 (2;17) | 4 (2;21) | 0.320 |
| Live-ventilator free days at day 28 |  | 145 | 28 (0;28) | 28 (0;28) | 28 (0;28) | 28 (0;28) | 0.943 |
| ECMO support |  | 145 | 2 (1.4) | 1 (2.0) | 1 (1.7) | 0 (0) | 1.000 |
| Vasopressor support |  | 145 | 41 (28.7) | 13 (26.0) | 19 (31.7) | 9 (27.3) | 0.817 |
| Renal replacement therapy |  | 145 | 15 (10.4) | 4 (8.0) | 7 (11.7) | 4 (11.8) | 0.787 |
| Ventilator-acquired pneumonia (among IMV) | | 46 | 20 (43.5) | 7 (43.8) | 8 (40.0) | 5 (50.0) | 0.873 |
| CAPA |  | 145 | 7 (4.9) | 5 (10.0) | 2 (3.3) | 0 (0) | 0.135 |
| Dexamethasone |  | 145 | 100 (80.0) | 38 (79.2) | 42 (80.8) | 20 (80.0) | 0.980 |
| Tocilizumab |  | 145 | 28 (22.6) | 14 (29.2) | 11 (21.6) | 3 (12.0) | 0.244 |
| Monoclonal antibodies |  | 145 | 16 (12.8) | 10 (20.8) | 2 (3.8) | 4 (16.0) | **0.034** |
| Casirivimab-Imdevimab |  | 145 | 1 (6.3) | 0 (0) | 0 (0) | 1 (25.0) | 0.375 |
| Tixagevimab-Cilgavimab |  | 145 | 15 (93.8) | 10 (100.0) | 2 (100.0) | 3 (75.0) | 0.375 |
| Sotrovimab |  | 145 | 0 | 0 | 0 | 0 | - |
| Duration of ICU stay, days |  | 145 |  |  |  |  |  |
| All patients |  | 145 | 9 (3;18) | 11 (5;22) | 7 (3;15) | 4 (2;21) | 0.320 |
| Survivors only |  | 110 | 7 (3;16) | 9 (5;22) | 6 (3;14) | 6 (3;15) | 0.084 |
| Day-28 mortality |  | 145 | 35 (24.5) | 11 (22.0) | 16 (26.7) | 8 (24.2) | 0.880 |

Results are N (%), means (±standard deviation) or medians (interquartile range); MV: mechanical ventilation; ECMO: extracorporeal mechanical ventilation; VAP: ventilator-acquired pneumonia; IMV: invasive mechanical ventilation; CAPA: COVID-19-associated pulmonary aspergillosis; ^a^ VAP episodes were recorded per definition in patients under IMV since more than 48 hours; Two-tailed p-values come from unadjusted comparisons using Chi square or Fisher’s exact tests for categorical variables, and t-tests or Mann-Whitney tests for continuous variables, as appropriate. No adjustment for multiple comparisons was performed; **Bolded** p-values are significant at the p<0.05 level.

Additional **Table S4.** Clinical and biological characteristics of the 145 patients with severe SARS-CoV-2 infection at the time of their intensive care unit admission according to their vital status at day 28.

|  | | **Day 28 survivors** | **Day 28 decedents** | **p-value** |
| --- | --- | --- | --- | --- |
|  |  | N=119 | N=37 |  |
| ***Demographics and comorbidities*** | |  |  |  |
| Sex, females |  | 38 (31.9) | 10 (27.0) | 0.572 |
| Age, years |  | 65.8 (±14.7) | 72.6 (±9.9) | **0.010** |
| Diabetes |  | 39 (33.6) | 13 (35.1) | 0.845 |
| Obesity |  | 34 (28.8) | 6 (16.02) | 0.127 |
| Chronic heart failure |  | 14 (12.1) | 4 (10.8) | 1.000 |
| Hypertension |  | 66 (56.9) | 27 (73.0) | 0.081 |
| Chronic respiratory failure |  | 26 (22.4) | 11 (30.6) | 0.320 |
| Chronic renal failure |  | 26 (22.4) | 7 (18.9) | 0.653 |
| Cirrhosis |  | 3 (2.6) | 0 (0) | 1.000 |
| Immunosuppression |  | 43 (37.1) | 20 (54.1) | 0.068 |
| Immunosuppression | None | 73 (62.9) | 17 (45.9) | **0.047** |
|  | Solid organ transplant | 15 (12.9) | 5 (13.5) | 0.538 |
|  | Onco-hematological malignancies | 10 (8.6) | 10 (27.0) | **0.005** |
|  | Others^a^ | 18 (15.5) | 5 (13.5) | 0.758 |
| Number of comorbidities |  | 2 (1;3) | 2 (2;3) | 0.250 |
| Clinical frailty scale |  | 3 (3;4) | 4 (3;4) | 0.274 |
| ***SARS-CoV-2 infection and Vaccination*** | |  |  |  |
| Omicron sublineage | |  |  | 0.880 |
| *BA.2* | | 39 (36.1) | 11 (29.7) | 0.851 |
| *BA.4/BA.5* | | 44 (40.7) | 16 (43.2) | 0.572 |
| *BQ.1.1 group* | | 36 (30.3) | 10 (27.0) | 0.812 |
| Previous SARS-CoV-2 infection | | 4 (3.4) | 4 (10.8) | 0.097 |
| SARS-CoV-2 vaccination | | 88 (74.6) | 27 (79.4) | 0.563 |
| Number of doses among vaccinated | | 3 (3;3) | 3 (3;3) | 0.857 |
| 3^rd^ dose- ICU admission^b^, days | | 163 (127;186) | 208 (163;254) | 0.951 |
| Last dose - ICU admission^b^, days | | 181 (78;263) | 208 (163;254) | 0.610 |
| SARS-CoV-2 serology at ICU admission | Unavailable | 59 (49.6) | 19 (51.4) | 0.514 |
|  | Negative^c^ | 15 (12.6) | 7 (13.1) |  |
|  | Positive | 45 (37.8) | 11 (29.7) |  |
| First symptoms - ICU admission, days | | 4 (2;9) | 2 (6;12) | 0.192 |
| SARS-CoV-2 RNA detection in nasopharyngeal swabs, Ct | | 20 (17;24) | 19 (15;22) | 0.054 |
| ***Patients severity upon ICU admission and biological features*** | |  |  |  |
| WHO 10-point scale |  | 6 (6;6) | 6 (6;8) | 0.151 |
| SAPS II score |  | 34 (27;43) | 37 (31;58) | **0.022** |
| SOFA score |  | 4 (3;6) | 5 (3;8) | **0.013** |
| PaO_2_/FiO_2_ ratio, mmHg |  | 158 (104;252) | 136 (89;167) | 0.125 |
| Arterial lactate level, mM |  | 1.5 (1.0;2.3) | 1.8 (1.0;3.9) | **0.007** |
| Blood leukocytes, G/L |  | 8.8 (6.3;12.4) | 10.7 (6.1;18.8) | **0.008** |
| Blood lymphocytes, G/L |  | 0.5 (0.3;0.8) | 0.4 (0.2;0.9) | **0.018** |
| Blood platelets, G/L |  | 175 (117;253) | 202 (158;274) | 0.147 |
| Serum urea level, mM |  | 8 (6;14) | 12 (8;18) | **0.024** |
| Serum creatinine level, µM |  | 99 (71;152) | 118 (85;230) | 0.077 |
| Bacterial coinfection |  | 31 (26.1) | 4 (10.8) | 0.070 |
| Oxygen/ventilatory support | Oxygen | 28 (23.9) | 6 (16.2) | 0.376 |
|  | High flow oxygen | 47 (40.2) | 15 (40.5) |  |
|  | NIV/C-PAP | 16 (13.7) | 3 (8.1) |  |
|  | Invasive MV | 26 (22.2) | 13 (35.1) |  |
| ECMO |  | 1 (0.8) | 0 (0) | 1.000 |
| Vasopressor support |  | 15 (12.6) | 13 (35.1) | **0.003** |

Results are N(%), means (±standard deviation) or medians (interquartile range). ^a^includes HIV infection, long-term corticosteroid treatment, and other immunosuppressive treatments; ^b^time lag between the last vaccination dose and ICU admission; ^c^defined as < 30 Binding Antibody Units (BAU)/mL; ICU: intensive care unit; Ct: cycle threshold; WHO: World Health Organization; SOFA: Sequential Organ Failure Assessment; SAPS II: Simplified Acute Physiology Score II; NIV: non-invasive ventilation; C-PAP; continuous-positive airway pressure; MV: mechanical ventilation; ECMO: extracorporeal mechanical ventilation; Two-tailed p-values come from unadjusted comparisons using Chi square or Fisher’s exact tests for categorical variables, and t-tests or Mann-Whitney tests for continuous variables, as appropriate. No adjustment for multiple comparisons was performed; **Bolded** p-values are significant at the p<0.05 level.
